# Awake Versus Asleep Intubation for Mediastinal Goiters: A Systematic Review and Meta-Analysis

**DOI:** 10.1177/19160216251333352

**Published:** 2025-05-30

**Authors:** Lindsay E. Booth, Norbert Banyi, Peter Rose, Shamir Karmali, Biljana Jonoska Stojkova, Donald W. Anderson, Oleksandr Butskiy

**Affiliations:** 1 Faculty of Medicine, University of British Columbia, Vancouver, BC, Canada; 2 Department of Anesthesiology, Pharmacology & Therapeutics, Faculty of Medicine, University of British Columbia, Vancouver, BC, Canada; 3 Department of Statistics, Faculty of Science, University of British Columbia, Vancouver, BC, Canada; 4 Division of Otolaryngology—Head and Neck Surgery, Department of Surgery, Faculty of Medicine, University of British Columbia, Vancouver, BC, Canada

**Keywords:** mediastinal goiter, thyroidectomy, awake intubation, asleep intubation, difficult airway management

## Abstract

**Importance:**

Mediastinal goiters can complicate anesthetic management, and although awake bronchoscopic intubation is the gold standard, it is resource-intensive and may be unpleasant for patients. In many centers across North America, patients undergoing thyroidectomy for mediastinal goiters are routinely intubated awake.

**Objective:**

This study aimed to evaluate the outcomes of intubation in patients selected for awake versus asleep intubation for thyroidectomy of mediastinal goiters.

**Design:**

PRISMA 2020 Checklist for systematic reviews was followed. A search was performed in the Medline, Embase, Web of Science, CINAHL, Scopus, and Cochrane databases. Two independent reviewers performed abstract and full-text review. Data were extracted in duplicate. Study quality was assessed using the JBI Critical Appraisal tool. To account for heterogeneity, a 3-level random-effects model was constructed using the Der Simonian and Laird method with an arcsine transformation.

**Setting and Participants:**

Patients undergoing thyroidectomy for benign mediastinal goiters.

**Intervention and Exposures:**

Awake or asleep intubation.

**Main Outcome(s) and Measure(s):**

Rate of failed intubations in asleep intubation and proportion of uncomplicated intubations in asleep and awake populations.

**Results:**

Twelve of 490 identified studies, involving 1002 patients, were included. Three cases of failed intubations were found in the asleep intubation group, with an overall incidence of failed intubation of 0.3%. Meta-analysis demonstrated an overall uncomplicated intubation rate of 91% (95% CI 77%-98%, n = 1002). Subgroup analyses showed a 96% success rate (95% CI 73%-100%, n = 60) for awake intubations and 88% (95% CI 69%-98%, n = 942) for asleep intubations. Further refined analyses showed uncomplicated intubation rates of 98% (95% CI 93%-100%, n = 469) for asleep, and 92% (95% CI 78%-99%, n = 48) for awake groups.

**Conclusions and Relevance:**

The risk of failed intubation in patients with mediastinal goiters remains low, and awake intubation may require more attempts than asleep intubation. Further research with standardized definitions of intubation difficulty is needed.

## Graphical abstract

**Graphical Abstract:**
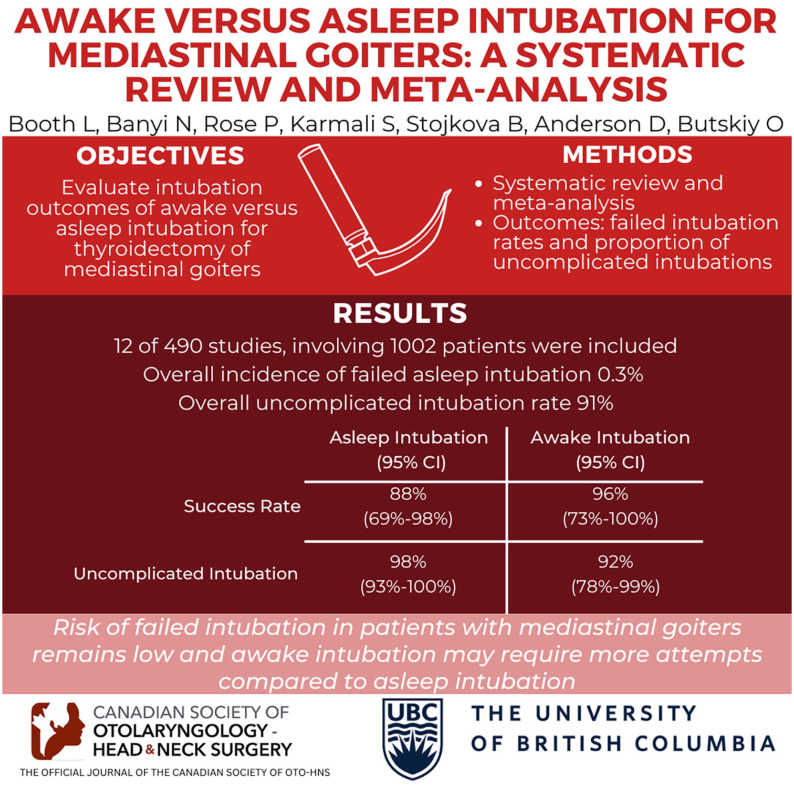

## Key Messages

- The overall incidence of failed intubation in the asleep population was 0.3% (3/942), none of which resulted in severe complications.
- There are higher rates of uncomplicated intubation in asleep intubation of patients with mediastinal goiter versus awake intubation.

## Introduction

Mediastinal goiters are defined as thyroid enlargement extending into the mediastinal space. They are also referred to as substernal, retrosternal, or intrathoracic goiters. Various definitions exist for mediastinal goiters, and there is controversy over the exact definition. In general, mediastinal goiters are defined as thyroid tissue extending below the level of the thoracic inlet with some definitions specifying that 50% of the tissue must be below the level of the thoracic inlet.^1^ Their prevalence has been estimated at 7% of all goiters.^2^ While some are asymptomatic, these goiters can cause compressive symptoms such as choking, dyspnea, or rarely-superior vena cava syndrome.^3^

The Canadian Airway Focus Group recommends video laryngoscopy as the first-line airway management in unconscious patients.^4^ However, awake tracheal intubation (ATI), often with a flexible bronchoscope, is recommended for anticipated difficult airways.^5^ ATI allows for the maintenance of spontaneous ventilation and airway protection. When combined with bronchoscopic guidance, this technique also confers the benefits of the scope acting as a guide for endotracheal tube (ETT) placement, and the ability to confirm ETT placement.^5^ In many centers, patients undergoing thyroidectomy for mediastinal goiters are routinely intubated using an awake technique.^6^ While ATI has a high rate of success when performed by an experienced operator, it is significantly-more time-consuming than asleep intubation and requires patient cooperation.^5^

Patients receiving head and neck surgeries are generally at a higher risk of difficult tracheal intubation than other surgical patients, due to pathology affecting the airway.^5^ In specific, mediastinal goiters have traditionally been thought to represent difficult airways due concerns of difficult bag and mask ventilation, tracheal intubation, and positive pressure ventilation attributed to tracheal compression and deviation.^7^ Between 35% and 73% of mediastinal goiters have some degree of tracheal compression.^8^ There is controversy over whether thyroidectomy for mediastinal goiters necessitates ATI or whether it can be successfully managed with asleep intubation. ATI confers the benefit of preventing “Can’t intubate, can’t oxygenate” scenarios. One previous review conducted by Bennett et al assessed the risk of difficult intubation and postoperative tracheomalacia with thyroidectomy for mediastinal goiters; however, they did not perform a systematic review, and few characteristics of the patient population and studies were included.^9^ As this review is now over 2 decades old, and numerous new studies have been published since, an updated review is warranted.^10-16^ The objective of this systematic review was to evaluate difficulty of intubation in patients selected for awake versus asleep intubation for thyroidectomy of mediastinal goiters.

## Methods

The protocol of this systematic review was reported in line with the Preferred Reporting Items for Systematic Reviews and Meta-Analyses (PRISMA) 2020 Checklist.^17^ The study protocol was registered with PROSPERO (ID: CRD42023393278).

### Search Strategy

Medline, Embase, Web of Science, CINAHL, Scopus, and the Cochrane database were searched. Results from database inception to search on February 6, 2023, were included. The search strategy was conducted in collaboration with a University of British Columbia library information specialist. The terms substernal goiter, mediastinal goiter, and intrathoracic goiter were utilized in conjunction with intubation and airway management including intubation and airway control. Forward and backward searches of references from included papers were performed by a single author (L.E.B.) and added to study screening. Covidence (Melbourne, Australia) automatically removed duplicate search results and was utilized for study screening, full-text review, and data extraction.

### Study Selection

Studies were included if their patient population had benign mediastinal goiters undergoing total, hemi-, or partial thyroidectomies with awake or asleep intubation and the paper reported on intubation techniques and outcomes. Primary research studies such as controlled trials, observational studies, or case series with greater than 3 patients were included. Case reports with 3 or fewer and review articles were excluded. Studies were excluded if they were reporting on patients with preexisting airway compromise secondary to pathology other than co-existing mediastinal goiter. Additionally, studies were excluded if the data were not extractable from the paper, if the study was not available in English, or if the duplicate data were used. In the case of duplicate data, the study with the larger sample size was included.

### Data Extraction

Data pertaining to patient characteristics, anesthetic characteristics, intubation method, intubation difficulty, and extubation difficulty were extracted in duplicate by 2 reviewers. Discrepancies were resolved through consensus. Uncomplicated intubations were defined by the research team as intubations with first pass success regardless of technique used. Complicated intubations were defined as intubations requiring greater than 1 intubation attempt. Failed intubation was defined as inability to intubate after 3 attempts or development of “cannot intubate, cannot oxygenate” scenarios. In the case of studies that defined intubation difficulty differently than our definition, we reviewed the definition and classified intubations as best possible according to our definitions. Where not explicitly described, intubations were assumed to be asleep. If no complications were reported, intubations were assumed to be uncomplicated.

### Risk of Bias Assessment

Study quality was assessed using the JBI Critical Appraisal tool for case series.^18^ Studies were rated independently by 2 reviewers (L.E.B. and N.B.), and discrepancies were settled through consensus. If consensus could not be reached, a third reviewer (O.B.) settled the discrepancy.

### Data Analysis

Data analysis was performed in R Studio (Version 4.2.3), using the packages metafor (version 4.4-0) and meta (version 6.5-0).^19,20^ Studies were weighted according to their sample size, and pooled outcomes were assessed. A 3-level random-effects model^21^ was constructed using the Der Simonian and Laird method with an arcsine transformation, with an estimate of heterogeneity being taken from the Mantel-Haenszel model. The *I*^2^ statistic was employed to assess interstudy heterogeneity, while a funnel plot was constructed to examine the general publication bias (Supplemental Figure 1). In cases where a quantitative analysis was deemed inappropriate, descriptive statistics were performed. Weighted means along with confidence intervals, informed by weighted standard deviations, were calculated where appropriate.

### Heterogeneity Analysis

Several strategies were used to reduce heterogeneity. We included only studies with both awake and asleep arms (*k* = 6), minimizing variation due to study type. Considering arms as independent but correlated within studies, we employed a 3-level random-effects model to measure heterogeneity within studies (between arms) and across studies. Despite these measures, heterogeneity remained high (QE [df = 10] = 475.5, *P* < .0001), prompting a sensitivity analysis. This involved applying the 3-level model to all the 2-arm studies, excluding one at a time, to identify the primary contributor. This analysis (Supplemental Table 1) pinpointed Sarı as the major contributor.^22^ However, removing this study still did not completely eliminate heterogeneity (QE [df = 8] = 70.5, *P* < .001).

## Results

### Search Results

**Figure 1.**
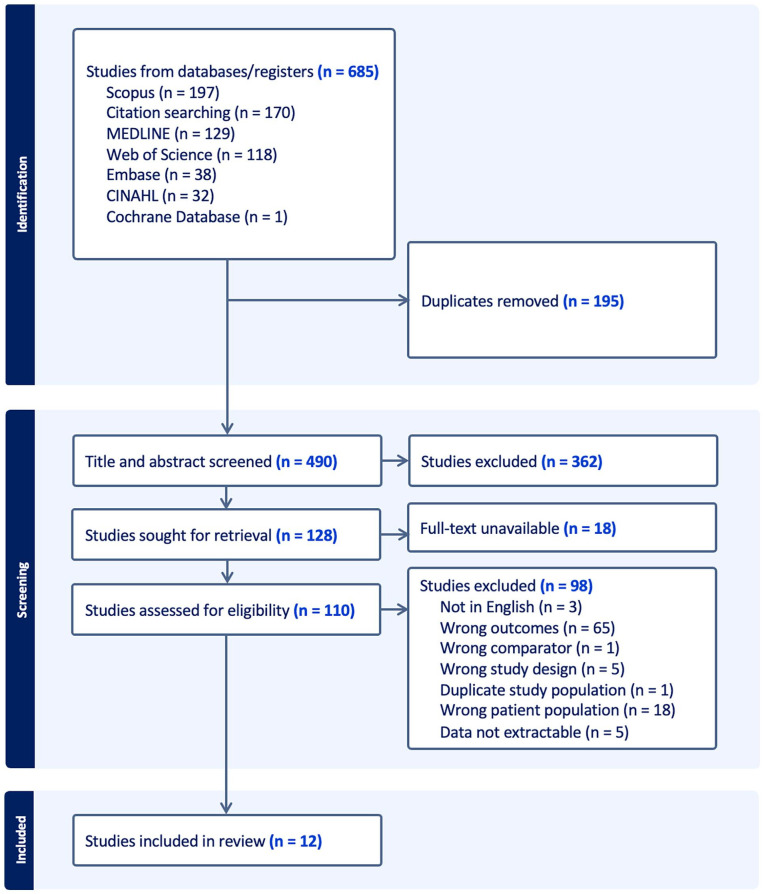
PRISMA diagram of the screening process.

Our search yielded 490 unique studies, of which 12 were included in the final analysis based on the inclusion criteria.^7,10-16,22-25^ The search results and review process are outlined in Figure 1. From the included studies, data pertaining to a total of 1002 patients were extracted. Seven of the 12 included studies were case series, whereas 5 were cohort studies. Of the 9 studies that reported on sex, 28% were male (268/959). From the 6 studies reporting on 761 patients, the mean age was 57.8 years old. A summary of study characteristics and raw data of the intubation method with respective outcomes can be found in Table 1.

**Table 1.** Summary of Study Characteristics and Outcomes of Awake Versus Asleep Intubation. The Following Definitions of Intubation Difficulty Were Used by the Research Team: Uncomplicated Intubations Were Defined as Intubations With First Pass Success Regardless of Technique Used. Complicated Intubations Were Defined as Intubations Requiring Greater Than 1 Intubation Attempt. Failed Intubation was Defined as Inability to Intubate After 3 Attempts or Development of “Cannot Intubate, Cannot Oxygenate” Scenarios.

Study characteristics						Awake				Asleep			
Study ID	Study design	Sample size	Country	Male sex, n	Mean age	Overall	Uncomplicated	Complicated	Failed	Overall	Uncomplicated	Complicated	Failed
Sarı 2012^22^	Case series	260	Turkey	70	57.4	12	12	0	0	248	83	165	0
Cohen 2009^11^	Case series	113	United States	29	56.9	7	7	0	0	106	106	0	0
Cho 1986^24^	Case series	70	United States	11		2	2	0	0	68	68	0	0
Cassai 2019^12^	Cohort study	57	Italy	15	63.3	0	0	0	0	57	44	13	0
Sajid 2017^13^	Case series	28	India	—	—	0	0	0	0	28	28	0	0
Bartın 2022^14^	Cohort study	106	Turkey	32	—	0	0	0	0	106	80	26	0
Tasche 2022^15^	Case series	179	United States	65	55.1	17	10	7	0	162	138	24	0
Pan 2020^16^	Case series	22	China	7	—	5	5	0	0	17	17	0	0
Amathieu 2006^25^	Cohort study	6	France	—	—	0	0	0	0	6	4	2	0
Dempsey 2013^7^	Case series	19	UK	11	65	0	0	0	0	19	18	0	1
Gilfillan 2014^10^	Cohort study	133	Australia	28	59.7	17	15	2	0	116	114	2	0
Olusomi 2018^23^	Cohort study	9	Nigeria	—	—	0	0	0	0	9	2	5	2
Total		1002				60	51	9	0	942	702	237	3

### Awake Intubation

Of 60 awake intubations, 9 (15%) were reported to be complicated. There were no cases of failed intubation; however, 2 of 32 (6.25%) reported instances of flexible endoscopy were converted to direct or video laryngoscopy.

### Asleep Intubation

Of 942 asleep intubations, 240 (25.5%) were reported to be complicated and there were 3 (0.3%) failed intubations whereby the patient received a tracheostomy which was subsequently removed.^7,23^ The outcomes of failed intubation for 2 patients described by Olusomi et al^23^ were not reported, whereas 1 patient from Dempsey et al^7^ presented again 16 months later and was successfully intubated using ATI, which revealed grade 3 laryngoscopy.

### Goiter Characteristics

Overall, 407 patients were reported to have tracheal deviation. Of the 126 patients who had tracheal deviation present and intubation outcome reported, 102 (81%) had uncomplicated intubation and 24 (19%) had a complicated intubation. There were no reports of tracheal deviation leading to intubation failure. Five studies examined thyroid gland weight and 4 examined goiter size, with 2 investigating their association with intubation difficulty.^7,10,11,14-16,22^ Tasche et al^15^ found no link between thyroid weight or goiter size and intubation difficulty, while Bartın et al^14^ reported that heavier thyroid glands were associated with more difficult intubation.

### Complications

Three of 942 (0.3%) patients who received asleep induction were reported to have failed intubation.^7,23^ Seven patients developed postoperative tracheomalacia. One patient required an emergency tracheostomy. One death was reported, due to surgical complications, in a patient who developed perioperative bleeding following the lysis of an adhesion between the mediastinal goiter and a large vessel within the thoracic cavity.^16^

### Meta-Analysis

**Figure 2.**
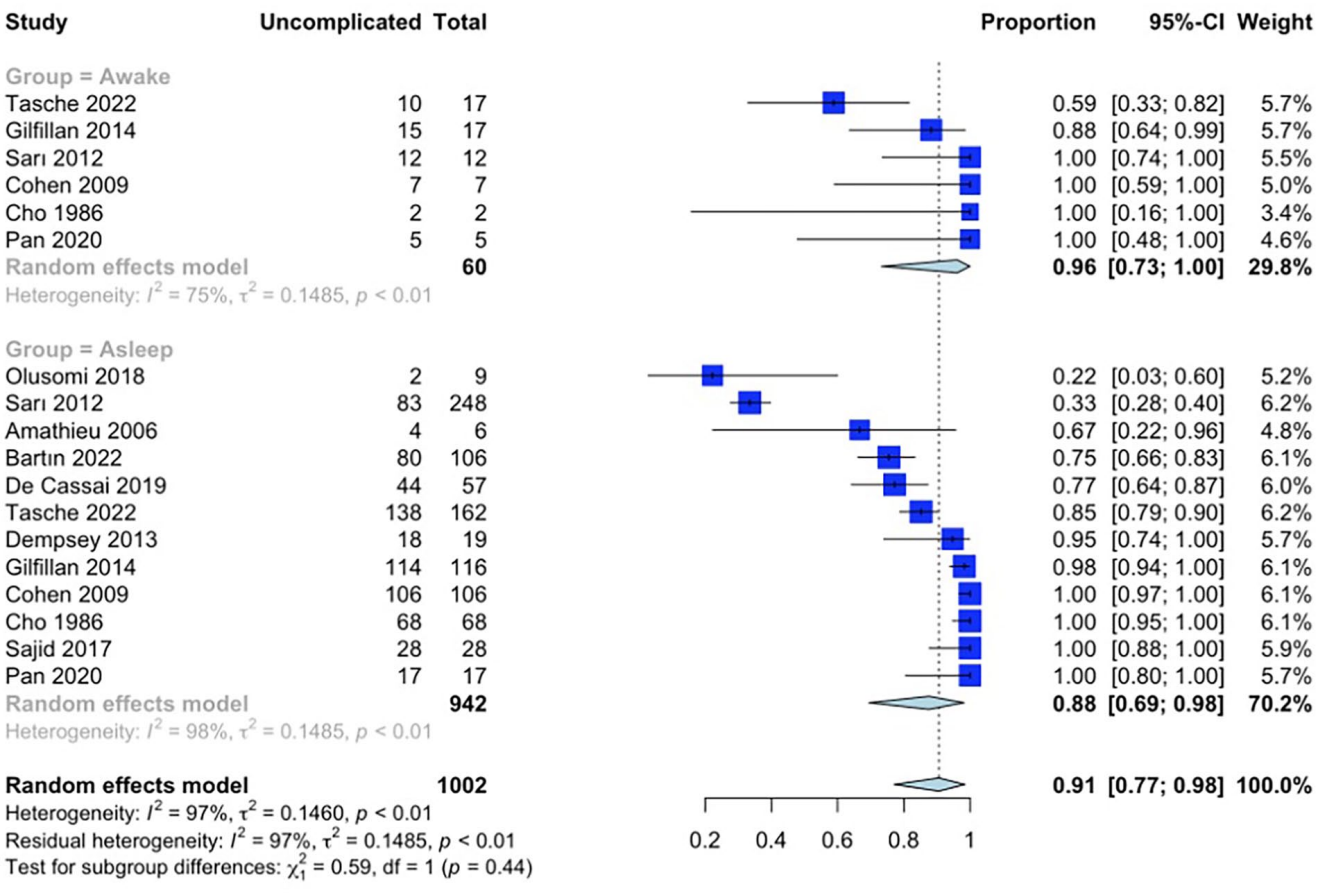
Meta-analysis with subgroup analysis comparing the proportion of uncomplicated intubations between awake and asleep intubations for all included studies.

**Figure 3.**
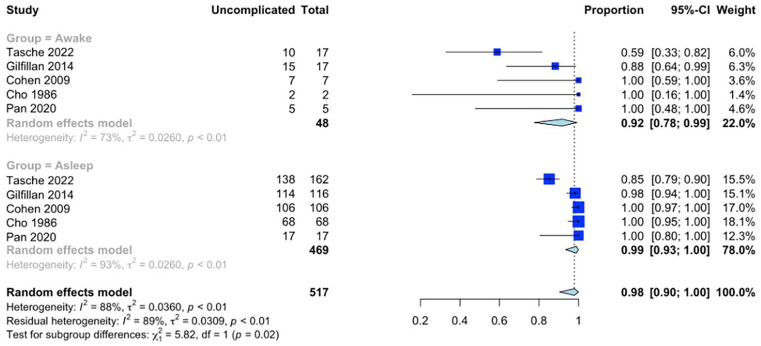
Meta-analysis results from the 3-level random effect model with subgroup analysis of studies examining the proportion of uncomplicated intubations between awake and asleep patients for included studies following statistical reduction in heterogeneity.

Meta-analysis of all 12 studies showed an overall 91% (95% CI 77%-98%, n = 1002) rate of uncomplicated intubation (Figure 2). On subgroup analysis of the awake and asleep groups, the rate of uncomplicated intubation was 96% (95% CI 73%-100%, n = 60) and 88% (95% CI 69%-98%, n = 942), respectively (Figure 2, *P* = .44) (between study heterogeneity estimate of *I*^2^ = 97%, tau^2^ = 0.146, *P* < .01). A subgroup analysis of 5 studies left after the exclusion of the 7 studies due to heterogeneity demonstrated the overall rate of uncomplicated intubation of 98% (95% CI 90%-100%, n = 517) (Figure 3). In addition, the uncomplicated intubation rates for awake and asleep intubation groups were of 92% (95% CI 78%-99%, n = 48) and 98% (95% CI 93%-100%, n = 469), respectively (Figure 3, *P* = .02) (between study heterogeneity estimate of *I*^2^ = 88%, tau^2^ = 0.036, *P* < .01).

### Risk of Bias

**Figure 4.**
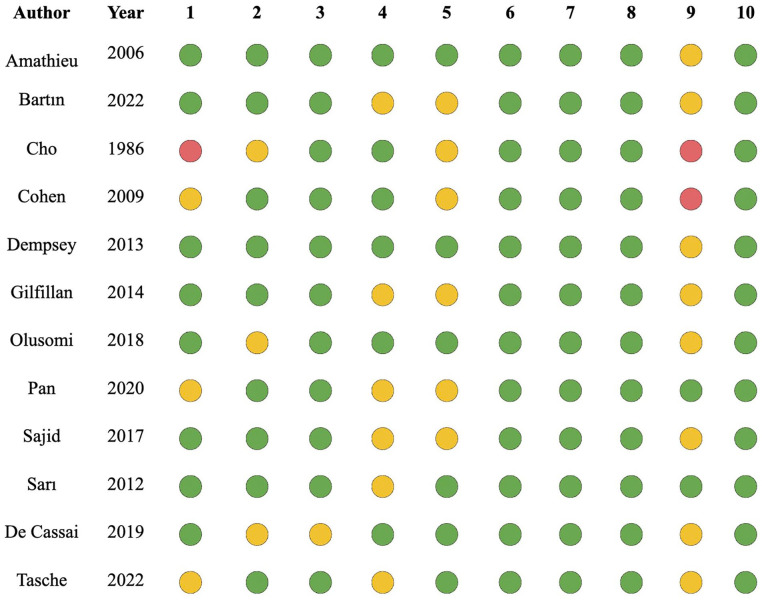
Risk of bias. The table can be interpreted as the following: green, demonstrated; yellow, unclear; red, not demonstrated. JBI case series criteria: (1) Clear criteria for inclusion? (2) Measured in a standard, reliable way for all participants included? (3) Valid methods used for identification of the condition for all participants? (4) Consecutive inclusion of participants? (5) Complete inclusion of participants? (6) Clear reporting of the demographics of the participants in the study? (7) Clear reporting of clinical information of the participants? (8) Outcomes or follow-up results of cases clearly reported? (9) Was there clear reporting of the presenting site(s)/clinic(s) demographic information? (10) Was statistical analysis appropriate?

The risk of bias collected for included studies are summarized in Figure 4. Studies on average met 7.5/10 of the JBI criteria. All studies had clear reporting of the demographics of the participants in the study, clear reporting of clinical information of the participants, and clear outcomes or follow-up results of cases. All studies except De Cassai (2019)^12^ clearly defined valid methods for identifying patients. Only 2 studies met criteria for describing presenting site(s)/clinic(s) demographic information.

## Discussion

This systematic review only found 3 explicit patient cases where intubation failed following asleep induction, representing only 0.3% of included cases (n = 1002). Two of these patients were from a study by Olusomi et al, where 9 of 125 consecutive thyroidectomy patients had mediastinal goiters and underwent asleep intubation via direct laryngoscopy at a single resource-challenged institution in Ilorin, Nigeria.^23^ In the case detailed by Dempsey (2013), the study was conducted at Aintree University Hospital in Liverpool, United Kingdom, and they describe that they were able to perform a tracheostomy and the patient fully recovered. However, any difficult intubation could potentially lead to severe adverse outcomes and large goiters may distort anatomy, making an emergency airway more difficult to achieve.^7^ The rate of complicated intubation varies widely depending on the definition of complicated intubation and study population.^26^ In our study, the overall rate of complicated intubation was 9%, which, as expected, is higher than the 5.8% (95% CI 4.5%-7.5%) reported by Shiga et al in their systematic review and meta-analysis of 35 studies involving 50,760 patients without airway pathologies.^27^ There is a lack of consensus in the literature on airway management for mediastinal goiters. In a 2011 study, a case of anesthetic management for a patient with tracheal compression secondary to mediastinal goiter who underwent emergent thyroidectomy was presented to a panel of 14 airway experts.^6^ The anesthetic plans presented by experts not only differed widely, but some experts unknowingly condemned plans recommended by other experts as unsafe.^6^

Our initial meta-analysis did not reveal a statistically-significant difference in the rate of uncomplicated intubations between patients undergoing awake versus asleep intubation (Figure 2, *P* = .44). However, after adjusting for heterogeneity, we observed that patients who underwent asleep intubation exhibited a statistically-significant higher incidence of uncomplicated intubations (Figure 3, *P* = .02). This is expected, as there is likely a bias due to the unmeasured confounding effect introduced by anesthesiologists using clinical judgment for awake or asleep intubation instead of randomization into each arm. Specifically, an awake technique is likely to be selected when a more difficult intubation is suspected as the patient is able to continue maintaining their own airway, thereby decreasing the risk of mediastinal airway obstruction on induction of anesthesia. While our results suggest that awake intubations are more likely to be complicated, failed intubations in this patient cohort are at lower risk of adverse outcomes than in patients receiving asleep intubation.

Another finding of our study was a high non-first pass success rate. In the asleep group, while 702 of 942 intubations were uncomplicated, 237 of 240 intubations that were complicated did not result in failure. Only 5 studies,^10,12,14-16^ with a median publication year of 2020, reported using video laryngoscopy in asleep patients and 3 reported using it to secure the airway in complicated intubations.^12,14,15^ The rest of the studies did not explicitly report utilizing video laryngoscopy and were older (median year of publication 2012), therefore unlikely to have utilized this method of visualization. A Cochrane review of 222 randomized control trials by Hansel et al demonstrated that hyperangulated videolaryngoscopy reduced rates of esophageal intubation and improved rates of successful intubation in patients with difficult airways.^28^ It is likely that such technologies would result in lower rates of complicated intubations and higher non-first pass success rates than what was found in our study.

It is important to interpret our data in the context of how studies defined complicated intubation. The authors of most studies included in our report either did not define complicated intubation or defined complicated intubations as intubations that deviated from original plans, took multiple attempts, or required nonstandard equipment. Only one study defined a “can’t intubate, can’t oxygenate” scenario as a complicated intubation, but did not encounter this issue.^10^ Therefore, despite encountering an overall complicated intubation rate of 12% in asleep patients, this does not imply that they were clinically-significant events. In specific, a considerable number of studies did not explicitly report instances of, or lack thereof, failed intubations or catastrophic outcomes within the asleep patient group, leaving unclear whether such events occurred. This represents a notable gap in the literature, underscoring the need for future studies to explicitly report the rate of failed intubations, even if none occurred, particularly in patients with challenging intubation conditions like mediastinal goiter. Furthermore, the literature demonstrated significant heterogeneity in the definition of complicated intubation, contributing to the persistent high heterogeneity observed in our meta-analysis (*I*^2^ = 88%), even after the exclusion of studies that most contributed to this heterogeneity. Furthermore, the observed heterogeneity may be induced by the uncontrolled and anecdotal nature of the case series,^29^ which constitute the majority of the studies included in this meta-analysis. To mitigate this issue, future research should adopt more standardized definitions of complicated intubation, such as employing the Intubation Difficulty Score as suggested by numerous studies.^23,25,30^ Additionally, a limitation of our study is that we were unable to account for the authors’ institutional experience and resources, which were difficult to obtain and likely contributed to the observed heterogeneity.

The review underscores the need for more detailed data as the current lack of specificity likely contributes to the widespread general use of fiberoptic intubation for patients with mediastinal goiters.^31^ For example, a significant portion of the studies did not describe varying anesthetic approaches within the mediastinal goiter patient population.^12-14,23,25^ Four of the 6 studies that had both patients undergoing asleep intubation and ATI did not describe the parameters used to choose whether awake or asleep intubations were done.^11,15,16,22^ Cho et al utilized awake intubations in 2 patients who were unable to lie supine and required nasotracheal intubation over a fiberoptic bronchoscope. In a study by Gilfillan et al, the reasons for suspecting difficult airway in patients who received ATI versus asleep intubations were reported, but in many cases, they were vague or not documented. Moreover, only two-thirds of included studies focused exclusively on patients with mediastinal goiters,^7,10,11,14-16,22,24^ whereas these patients constituted only a subset within broader study populations. Moreover, there was inconsistent reporting of the association between goiter characteristics and intubation difficulty, limiting the conclusions that could be drawn in our study. Future studies should aim to define which subpopulations of patients with mediastinal goiters require ATI and which do not, taking into consideration patient factors such as goiter characteristics.

## Conclusion

Failed asleep intubation in patients with mediastinal goiters is rare and has been reported in 0.3% of all cases in the included literature. For patients undergoing thyroidectomy for mediastinal goiters, asleep intubation as compared to awake intubation may represent an increased risk for complicated intubation, defined as intubation requiring more than one attempt. Further studies are needed and should employ standardized definitions of intubation difficulty.

## Supplemental Material

sj-docx-1-ohn-10.1177_19160216251333352 – Supplemental material for Awake Versus Asleep Intubation for Mediastinal Goiters: A Systematic Review and Meta-Analysis Supplemental material, sj-docx-1-ohn-10.1177_19160216251333352 for Awake Versus Asleep Intubation for Mediastinal Goiters: A Systematic Review and Meta-Analysis by Lindsay E. Booth, Norbert Banyi, Peter Rose, Shamir Karmali, Biljana Jonoska Stojkova, Donald W. Anderson and Oleksandr Butskiy in Journal of Otolaryngology - Head & Neck Surgery

sj-png-2-ohn-10.1177_19160216251333352 – Supplemental material for Awake Versus Asleep Intubation for Mediastinal Goiters: A Systematic Review and Meta-Analysis Supplemental material, sj-png-2-ohn-10.1177_19160216251333352 for Awake Versus Asleep Intubation for Mediastinal Goiters: A Systematic Review and Meta-Analysis by Lindsay E. Booth, Norbert Banyi, Peter Rose, Shamir Karmali, Biljana Jonoska Stojkova, Donald W. Anderson and Oleksandr Butskiy in Journal of Otolaryngology - Head & Neck Surgery
