# Supplementary material for: Awake Versus Asleep Intubation for Mediastinal Goiters: A Systematic Review and Meta-Analysis
Source: J Otolaryngol Head Neck Surg. 2025 May 30;54:19160216251333352. doi: 10.1177/19160216251333352 (PMC12125518; doi:10.1177/19160216251333352)
Supplement: sj-docx-1-ohn-10.1177_19160216251333352 – Supplemental material for Awake Versus Asleep Intubation for Mediastinal Goiters: A Systematic Review and Meta-Analysis [file sj-docx-1-ohn-10.1177_19160216251333352.docx]

**Suppl. Figure 1:** Funnel plots of residuals for studies before (A) and after (B) heterogeneity analysis.

**Suppl. Table 1**. Results of sensitivity analysis. QE (residual heterogeneity) is significantly reduced with the removal of Sarı 2012 as compared to other studies. P-values and SE of asleep and awake estimates are reported on arcsine transformed scale. The 95% confidence intervals were constructed on the arcsine scale, and then back-transformed to the original scale along with the overall estimates of the proportions for each arm.

| Study Removed | QE  (df =8, p < 0.001) | Asleep Estimate (SE, CI, p) | Awake Estimate (SE, CI, p) |
| --- | --- | --- | --- |
| Sarı 2012 | 70.5 | 0.99 (0.14, 0.86 - 0.97, <0.001) | 0.92 (0.18, 0.64-1.00, 0.31) |
| Cohen 2009 | 376.2 | 0.91 (0.24, 0.50 - 0.97, < 0.001) | 0.94 (0.25, 0.55-0.94, 0.80), |
| Cho 1986 | 416.5 | 0.91 (0.24, 0.50 - 0.97, <0.001) | 0.94 (0.25, 0.55-0.94, 0.81) |
| Tasche 2022 | 458.6 | 0.94 (0.23, 0.59 - 0.95, <0.001) | 1.00 (0.23, 0.78-1.00, 0.38) |
| Pan 2020 | 460.1 | 0.91 (0.24, 0.50 - 0.97, <0.001) | 0.94 (0.25, 0.55-0.94, 0.81) |
| Gilfillan 2014 | 422.5 | 0.92 (0.25, 0.51 - 0.96, <0.001) | 0.98 (0.26, 0.65-0.98, 0.53) |
