# Supplementary figures and images for: Awake Versus Asleep Intubation for Mediastinal Goiters: A Systematic Review and Meta-Analysis
Source: J Otolaryngol Head Neck Surg. 2025 May 30;54:19160216251333352. doi: 10.1177/19160216251333352 (PMC12125518; doi:10.1177/19160216251333352)

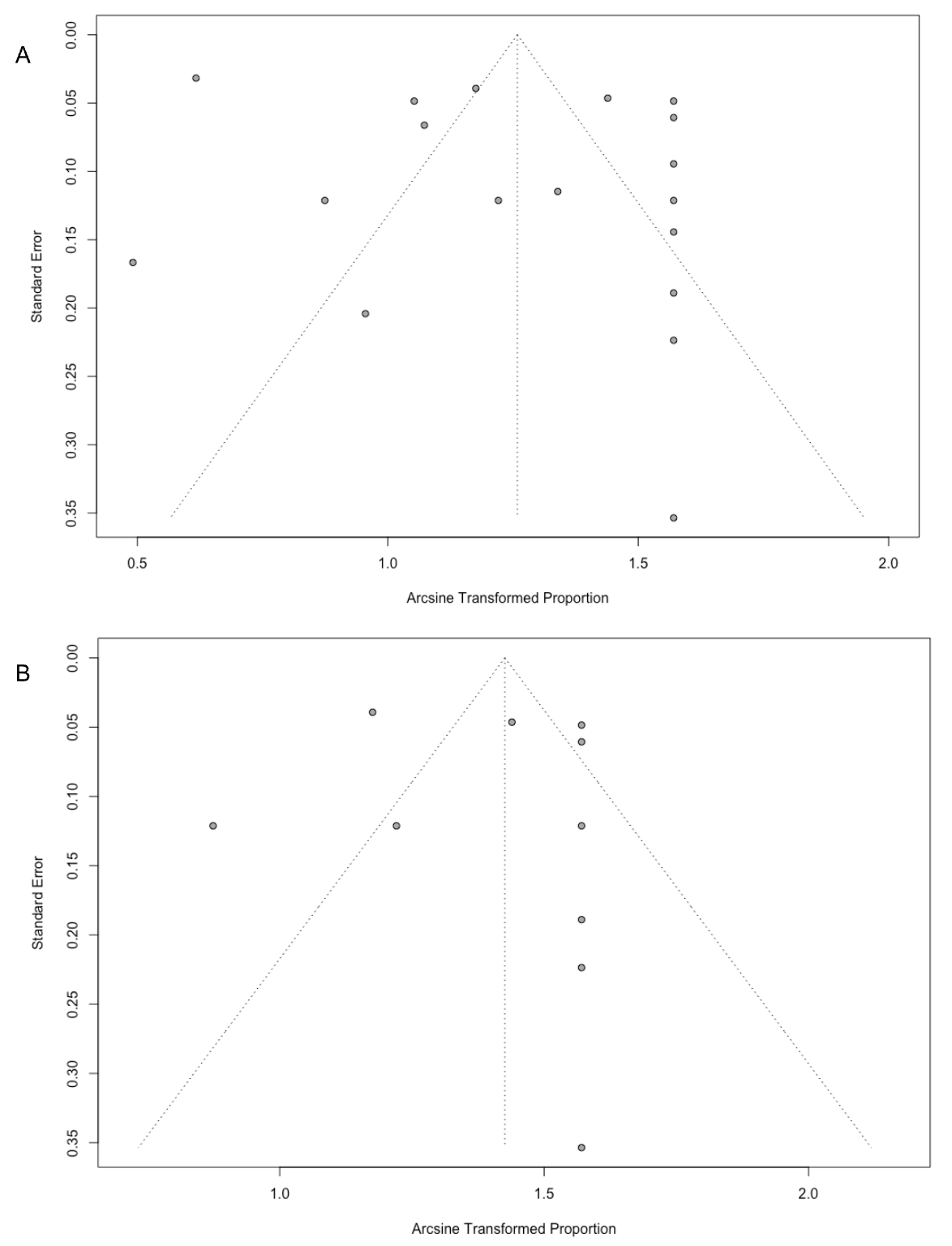

Supplement: sj-png-2-ohn-10.1177_19160216251333352 – Supplemental material for Awake Versus Asleep Intubation for Mediastinal Goiters: A Systematic Review and Meta-Analysis [file sj-png-2-ohn-10.1177_19160216251333352.png]
